# Pinocembrin ameliorates intermittent hypoxia-induced neuroinflammation through BNIP3-dependent mitophagy in a murine model of sleep apnea

**DOI:** 10.1186/s12974-020-02014-w

**Published:** 2020-11-11

**Authors:** Lin-Jing Gong, Xin-Yuan Wang, Wen-Yu Gu, Xu Wu

**Affiliations:** 1 Department of Pulmonary Medicine, Zhongshan Hospital, Fudan University, 180 Feng Lin Rd, Shanghai, 200032 China; 2 Department of Orthopaedics, Zhongshan Hospital, Fudan University, 180 Feng Lin Rd, Shanghai, 200032 China; 3 Department of Urology, Shanghai Tenth People’s Hospital, Tongji University School of Medicine, No. 301, Yanchang Rd, Shanghai, 200072 China

**Keywords:** Pinocembrin, Neuroinflammation, Microglia, Obstructive sleep apnea (OSA), BNIP3, Mitophagy

## Abstract

**Background:**

Intermittent hypoxia (IH) caused by obstructive sleep apnea (OSA) leads to neuroinflammation. Pinocembrin has been shown to have neuroprotective effects, while the therapeutic functions under IH condition are still unknown.

**Methods:**

An OSA model was established by CIH exposure inside custom-made chambers. C57BL/6 mice were intraperitoneally injected with pinocembrin (40 mg/kg, i.p.) or vehicle (PBS containing 5% povidone; i.p.), and the changes of behavior on mice were detected by the Morris water maze test. Immunohistochemical staining, western blotting, immunofluorescence assays, and immunoprecipitation were used to investigate the association between NLRP3 inflammasome and BNIP3-dependent mitophagy. The mitochondrial morphology and mitophagosomes were detected under a transmission electron microscope. The detrimental effects of IH were tested by annexin V-FITC/PI staining, Mito SOX Red staining, and JC-1 mitochondrial membrane potential assay.

**Results:**

In this study, our observations in vivo indicated that the administration of pinocembrin can restore spatial learning and memory ability and reduce neuronal apoptosis and hippocampal inflammation. Pinocembrin treatment significantly inhibited the formation of NLRP3 inflammasome and infiltration of microglia and enhanced BNIP3-mediated mitophagy in the hippocampus of IH mice. Additionally, our in vitro results show that pinocembrin protects microglial cells against IH-induced cytotoxicity by activating BNIP3-dependent mitophagy through the JNK-ERK signaling pathway.

**Conclusions:**

In summary, our findings demonstrated that pinocembrin can act as a potential therapeutic strategy for IH-induced neuroinflammation.

**Supplementary Information:**

The online version contains supplementary material available at 10.1186/s12974-020-02014-w.

## Introduction

Obstructive sleep apnea (OSA) is a breathing disorder characterized by recurrent episodes of upper-airway collapse, resulting in intermittent hypoxia (IH) during sleep [1, 2]. IH has been shown to induce neuroinflammation and subsequent behavioral and neuropsychological deficits in the central nervous system (CNS) [2]. A number of studies have demonstrated that IH triggers neuroinflammation through multiple signaling pathways, especially via oxidative stress, mitochondrial dysfunction, and an imbalance in pro- and anti-apoptotic gene cascades [3]. However, the underlying mechanisms are complex and incompletely understood. Microglia are the resident macrophages in the CNS and are the principal sources of pro-inflammatory factors in CNS affected by OSA [4, 5]. In recent years, accumulating evidence has implicated activated microglial cells in both the development and maintenance of neuroinflammation in patients with OSA [6, 7]. Therefore, the inhibition of the abnormal activation of microglial and the protection of neurons from neuroinflammation are potential therapeutic strategies for OSA-related neurocognitive impairment.

Pinocembrin (5,7-dihydroxyflavone) is a natural flavonoid drug that can cross the blood-brain barrier, having antimicrobial, anti-inflammatory, antioxidant, and anticancer properties [8, 9]. Notably, several studies have shown that pinocembrin also has a neuroprotective effect, for which possible mechanisms may include reversed autophagic activity and inhibited cell apoptosis [10]. These characteristics make pinocembrin an attractive molecule for the potential treatment of neuroinflammation in CNS diseases. Pinocembrin exerts its inhibitory effects against microglia activation by suppressing the phosphoinositide 3-kinase (PI3K)-Akt-nuclear factor (NF)-κB signaling pathway in lipopolysaccharide (LPS)-treated BV2 cells [11]. Xi et al. also found that pinocembrin restricts M1 microglial polarization by inhibiting toll-like receptor 4 (TLR4), thus playing a protective role against intracranial hemorrhage (ICH) injury [12]. Therefore, in the search to identify a neuroprotective agent that alleviates neuroinflammation, pinocembrin could provide unique functions in reducing reactive oxygen species (ROS) and modulating autophagy. However, the role of pinocembrin in the context of IH so far remains unclear.

Recent studies have shown that mitochondrial damage is involved in the activation of the nucleotide-binding domain-like receptor protein 3 (NLRP3) inflammasome via mitochondrial ROS (mtROS) production [13]. In our previous study, we highlighted the significant implications of NLRP3 in IH-induced mitochondrial damage [14]. mtROS accumulation is also the major trigger of the NLRP3-mediated inflammatory response, which eventually promotes the maturation of pro-inflammatory cytokines such as interleukin (IL)-1β [10]. Mitophagy, a type of selective autophagy, is traditionally recognized as a specific process that initiates the degradation of superfluous and damaged mitochondria to maintain mitochondrial homeostasis [15]. However, if this procedure is not properly activated, superabundant inflammation induced by NLRP3 can be harmful to the host [16]. In addition, the repressive autophagic activity during the activation of the NLRP3 inflammasome is attributed to compromised mitophagy [17].

BCL2-interacting protein 3 (BNIP3), primarily localized on the mitochondrium, is a potent and well-documented inducer of autophagy in many different cell types [1, 18, 19]. Through the Parkin-independent pathway of mitophagy, activated Bcl-2 and BNIP3 (or BNIP3-like, BNIP3/Nix) bind directly to LC3 to promote autophagic activity in a protective response against Bnip3 death signaling, mtROS overproduction, and NLRP3 inflammasome activation in CNS diseases [17, 20]. BNIP3-mediated mitophagy has been reported to protect against neuroinflammation [21]. Additionally, Lei et al. showed that BNIP3-mediated mitophagy has beneficial effects on intracellular homeostasis and reduces microglial apoptosis in the tumor necrosis factor α (TNF-α)-treated BV2 cells [22]. However, the association between the BNIP3-dependent mitophagy and the neuroprotective effect of pinocembrin is still unknown.

In this study, we demonstrate that pinocembrin improves behavior and cognitive deficits in IH mice via ameliorating microglia-induced neuroinflammation. Meanwhile, pinocembrin activates BNIP3-mediated mitophagy and inhibits NLRP3 inflammasome formation in hippocampal tissues in vivo. In the in vitro experiments, we show that pinocembrin prevents IH-elicited NLRP3 inflammasome activation in microglia, at least in part by enhancing mitophagy and attenuating mtROS production, which are mediated by the c-Jun N-terminal kinase (JNK)-extracellular regulated kinase (ERK)-BNIP3 signaling pathway. These data may provide a basis for using pinocembrin as a therapeutic intervention for OSA-associated neuroinflammation.

## Methods and materials

### Animals, cells, short hairpin RNA, antibodies, and reagents

The male C57BL/6J mice (6–7 weeks old, 20–22 g, *n* = 48) supplied by the Laboratory Animal Center at Fudan University (Shanghai, China) were raised in specific pathogen-free conditions. The murine microglial cell line BV-2 was obtained from the Chinese Academy of Medical Sciences (Beijing, China). We obtained pinocembrin (S3941) and 3-methyladenine (3-MA, S2767) from Selleck Chemicals (Houston, TX). Phosphatase inhibitor (P1045), SP600125 (S1876), and FR 108204 (SD5978) were obtained from Beyotime (Shanghai, China). We obtained ionized calcium-binding adaptor molecule 1 (Iba-1, ab65828), BNIP3 (ab10433), LC3 (ab51520), Beclin-1 (ab62557), NLRP3 (ab214185, ab4207 for immunofluorescence), Caspase-1 (ab1872), and Bax (ab32503) from Abcam; ATG7 (#8558), ATG5 (#12994), BNIP3/Nix (#12396), ASC (#67824), Caspase-3 (#9662), Bcl-2 (#3498), TOM20 (#42406, for western blot), and ubiquitin (#3933) from Cell Signal Technology (CST); TOM20 (AF1717, for immunofluorescence) from Beyotime; and Casase-1 p20 (sc-398715) from Santa Cruz Biotechnology. Fluorescence secondary antibodies were obtained from Jackson ImmunoResearch: goat anti-mouse IgG (Alexa Fluor® 555, 115-545-166), goat anti-rabbit IgG (Alexa Fluor® 488, 111-605-144), and donkey anti-goat IgG (Alexa Fluor® 555, 705-116-147). MitoSOX (M36008) was obtained from Thermo Fisher Scientific, and Cell Counting Kit-8 (CCK-8, CK04) was obtained from Solarbio® Life Science. Bnip3 small hairpin RNA (shRNA) plasmid was synthesized by GenePharma (Shanghai, China). The sequence of shRNA was as follows: 5′-GCCTCCGTCTCTATTTATAAT-3′.

### Establishment of the IH animal model and drug treatment

**Fig. 1:**
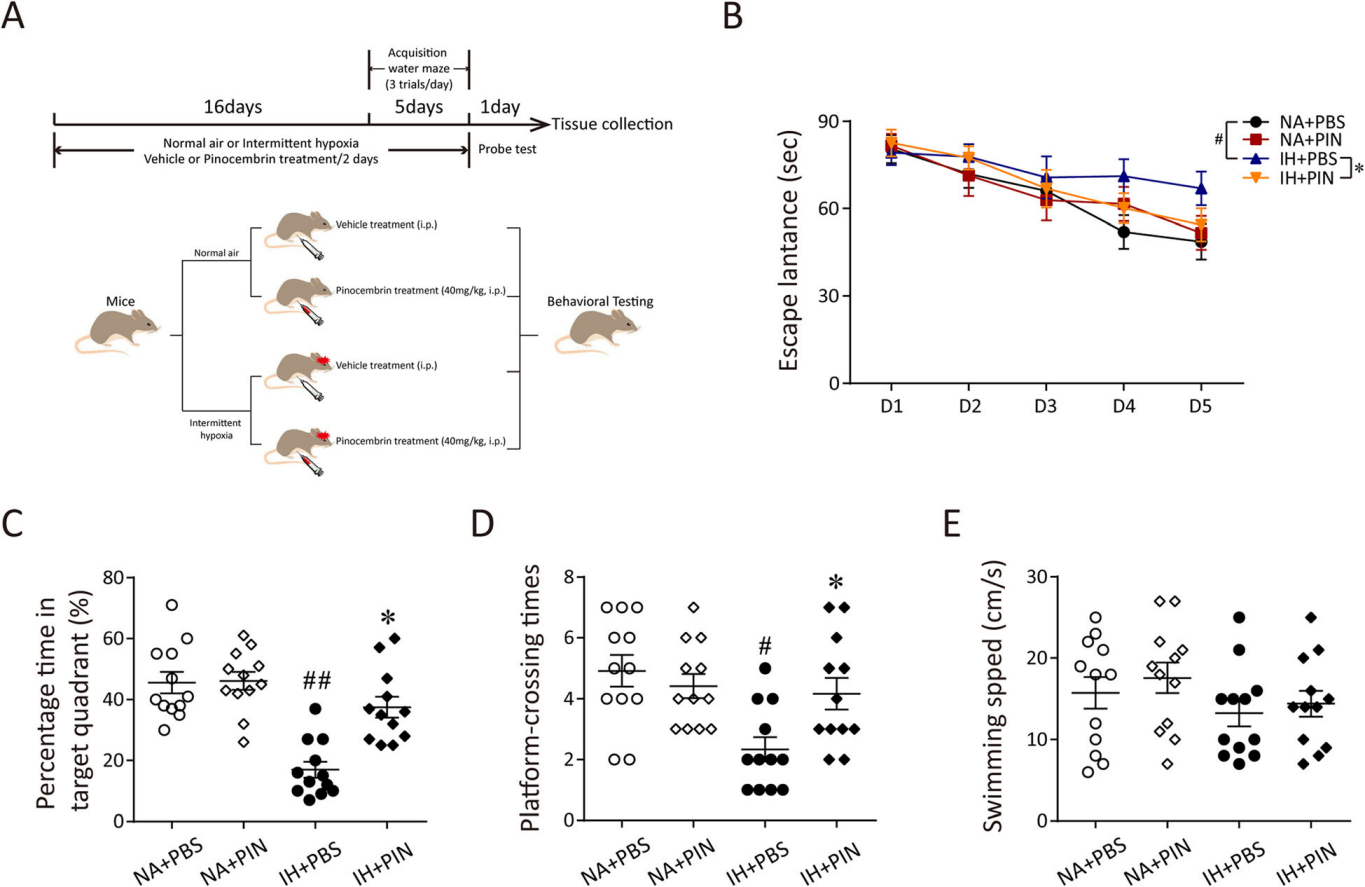
Pinocembrin treatment alleviates IH-induced learning and memory function deficits in mice. **a** The experimental protocols of IH treatment and behavioral tests. **b** Escape latency to reach the target platform during 5-day training in mice either exposed to NA or IH and receiving either vehicle or pinocembrin. **c** Percentage time in the target quadrant during probe test after completion of water maze training. **d**, **e** Average platform-crossing times and swimming speed in mice on the 6th day. All results are mean ± SEM (*n* = 12). ^#^*p* < 0.05 vs. the NA group; ^##^*p* < 0.01 vs. the NA group; *p < 0.05 vs. the IH group

A total of 48 mice were randomly divided into 4 groups of twelve: the normal air group (NA), the group treated with pinocembrin during 21 days of NA (NA + PIN), the 21-day IH group (IH), and the group treated with pinocembrin during 21 days of IH (IH + PIN). The mice from the PIN group were injected with 40 mg/kg pinocembrin every 2 days at 8:00 AM from day 1 to day 21 during the experiment (dissolved in PBS containing 5% povidone; i.p.). The mice from the NA and IH groups were injected with the vehicle (PBS containing 5% povidone; i.p.). The dosing, treatment regimens, and delivery route were based on previous work [23]. Mice were maintained in 4 specially designed chambers (28.5 × 30.0 × 51.5 cm) under standard conditions (23–25 °C, 50–60% humidity, 12-h light/dark cycle) with regular chow and water. The animals were acclimatized for 1 week followed with 21-day IH or NA treatment. IH was administered for 8 h/day, from 8:00 AM to 4:00 PM (when these animals typically sleep), with the oxygen level oscillating between 21 and 5% with a period of 60 s. The oxygen concentration was automatically measured using an oxygen analyzer (Corporation, Shanghai, China) and was changed by a computerized system controlling gas outlets of oxygen and nitrogen, as previously described [14]. Behavioral testing occurred during the final 5 days in IH or NA treatment during the dark phase and continued after the cessation of treatment (Fig. 1a). All the experimental protocols were approved by the Fudan University institutional animal care and use committee in accordance with the Helsinki Declaration of 1975.

### Morris water maze

The Morris water maze (MWM) was used to assess spatial reference learning and memory of mice as previously described [24]. The MWM consisted of a 1-m diameter circular pool, 0.6 m in height, filled to a level of 35 cm with water maintained at a temperature of 24 ± 2 °C. One hundred fifty milliliters of nontoxic white tempera paint was added to make pool water opaque. An escape platform was located in one of the quadrants of the pool and positioned 1 cm below the water surface. Mice were placed in other three different quadrants in turn and then the time taken (escape latency) for the mouse to find the escape platform was recorded. The mice were trained in 5 consecutive days, 3 trials per day with a 3-min inter-trial interval (ITI) for each mouse from 5:00 to 10:00 PM. After being placed into the pool, each mouse was allowed a maximum of 90 s to climb on the platform and then guided to the platform if they failed to find it. The position of the platform kept constant during the trials. Mice acquired spatial memory about the location of the escaped platform through these training sessions. The spatial memory of mice was tested 24 h later after the last training with the platform removed. To access the performance of mice in MWM, the percentage of time traveled in the target quadrant, platform-crossing times, and the average swimming speed on the sixth day were also recorded and analyzed.

### Establishment of the IH microglial cell model and drug treatment

BV2 were cultured in DMEM medium (Hyclone), containing 10% heat-inactivated fetal bovine serum (FBS), 100 U/mL penicillin, and 100 mg/mL streptomycin in a 37 °C incubator with 5% CO_2_ until 50–60% confluent. The method of IH-treated BV2 cells was completed as previously [14]. Briefly, cells were maintained in a 37 °C custom-made chamber with 5% CO_2_, in which O_2_ concentration was alternated between 0 and 22% every 30 min via injecting nitrogen or oxygen. The dissolved O_2_ was monitored by a laser O_2_ probe (Biospherix). The IH reached to 21% O_2_ and 5% O_2_ as normoxic and hypoxic values sensed by the cells. Transient transfections of BV2 cells with shRNA were performed by Lipofectamine™ 2000 (Invitrogen) according to the manufacturer’s instructions. Cells were cultured in 10% FBS DMEM medium for 48 h following 6-h transfection. Cells were pretreated with SP600125 or FR 108204 for 30 min before the administration of pinocembrin. 3-Methyladenine (3MA, 5 mM) was added to the culture medium for 6 h before exposing to IH.

Pinocembrin (purity > 99%) was dissolved in dimethylsulfoxide (DMSO) to make a 50-mM stock solution. We found that pinocembrin at doses of 20 μM could reduce cell viability (Fig. 4a). Therefore, we pretreated microglia with 1, 3, or 10 μM pinocembrin for 1 h before exposing them with IH. After exposurement, the cells were collected for flow cytometry analysis, immunofluorescent staining, qRT-PCR, or western blotting analysis. All experiments were performed in triplicate.

### Histopathology and immunohistochemistry

After the behavior test, mice (5/group) were anesthetized with isoflurane. Half of the hemispheres (right) were removed and post-fixed in 4% paraformaldehyde (PFA) for 24 h at 4 °C. After dehydration, brain hemispheres were embedded in paraffin and then cut into 4-μm-thick sections. To assess pathological analysis, hematoxylin and eosin (H&E) staining was performed. For immunohistochemistry, slices from hippocampi were used for staining with anti-NLRP3 (1:500) overnight at 4 °C. The next day, the brain slices were incubated with specific IHC detection reagent for 2 h at 37 °C. Images were obtained with a light microscope (Olympus/DP70, Tokyo, Japan).

### Immunofluorescence staining

Mice (5/group) were anesthetized with isoflurane and sacrificed following behavior test. The whole brain tissues were extracted immediately and cut sagittally into hemispheres. Left hemispheres were fixed in ice-cold 4% PFA overnight at 4 °C and were then equilibrated in 30% sucrose. Next, 25-μm sagittal slices in the hippocampi were obtained with a freezing microtome. Sections were washed in cold PBS three times, blocked with 5% goat serum for 1 h, and incubated with Iba-1 antibody (1:500) and BNIP3 antibody (1:200) overnight at 4 °C.

BV2 microglial cells were cultivated on cell slides for staining. Cells were fixed by 4% paraformaldehyde for 30 min at 37 °C and then permeated cell membrane using 0.1% Triton X-100 for 5 min at room temperature. Subsequently, the cell slides were incubated with LC3 (1:500), BNIP3 (1:200), TOM20 (1:200), NLRP3 (1:500), or ASC (1:500) antibodies at 4 °C overnight. After incubating with secondary antibody for 1 h at room temperature, all the slices were counterstained with DAPI (Vector Laboratories, CA, USA), and photomicrographs were captured using an Olympus DP70 digital camera and software (Olympus, Japan) or a confocal microscope (Nikon, Japan). Samples were analyzed in a blinded manner using 3–5 fields randomly selected from each group. The co-localization analysis was performed by the ImageJ software.

### RNA isolation and quantitative reverse transcriptase-PCR (qRT-PCR)

Total RNA was extracted from hippocampal lysates of mice (3/group) and BV2 cells using TRIzol reagent (Invitrogen, Carlsbad, CA). Relative mRNA levels were quantified by SYBR-Green Master Mix Plus (Toyobo, Osaka, Japan) according to the manufacturer’s protocol using a real-time PCR system (Applied Biosystems 7500HT; Applied Biosystems, Foster City, CA). The expressions of mRNA for TNF-α, iNOS, COX-2, IL-6, and IL-1β were performed in triplicate, and the samples were normalized by evaluating the GAPDH mRNA level. The primers specific to each mRNA used for the amplification were purchased from Sangon Biotech (Shanghai, China) and are listed in the supplementary material, Table S1.

### Immunoblot analysis

For western blot analysis, the proteins from hippocampal tissues (4/group), BV2 cells, and mitochondrial fraction were separated in 10% or 12.5% SDS-polyacrylamide gel, and polyvinylidene difluoride (PVDF) membranes were incubated with specific primary antibodies at 4 °C overnight. Bands were detected by ECL (Amersham Pharmacia Biotech, Piscataway, NJ) following 1-h incubation with HRP-conjugated secondary antibody (1:5000) at room temperature. ImageJ software (NIH, Bethesda, MD) was used for densitometry analysis, and the relative protein expressions were normalized against GAPDH or TOM20 (mitochondrial fraction, mito.). All experiments were performed in triplicate.

### Analysis of apoptosis

Apoptotic neuron was detected using terminal deoxynucleotidyl transferase dUTP nick-end labeling (TUNEL) staining according to the manufacturer’s protocol (in situ cell death detection kit; Roche, Netley, NJ). The number of TUNEL-positive cells was counted in a blinded manner using 5 individual images.

BV2 cells were collected and washed with PBS twice, and then cell apoptosis was assessed by flow cytometry and immunoblot analysis of caspase-3, Bcl-2, and Bax. Flow cytometry was performed by labeling annexin V-fluorescein isothiocyanate (FITC) and propidine iodide (PI) according to the manufacturer’s instructions (BD Biosciences, 556547). Cell mortality was analyzed by BD LSR Fortessa, and quantification analysis was conducted by FlowJo software (Tree Star, San Carlos, CA). The upper and lower right quadrants show the proportions of the late (Annexin V+/PI+) and the early (Annexin V+/PI−) apoptotic cells, respectively.

### Isolation of mitochondria

Microglial mitochondria were isolated and purified using the Cell Mitochondria Isolation Kit (Beyotime Biotech, C3601) as per the manufacturer’s protocol. Cell pellets (5 × 10^6^) were resuspended in 200 μl of Mitochondria Isolate Reagent after washing twice with cold PBS, incubated on ice, and then homogenized with a glass homogenizer. The homogenate was centrifuged at 600*g* for 10 min. The supernatant was transferred to a new tube and spun at 11,000*g* for 10 min at 4 °C to pellet mitochondria. The mitochondrial pellet was washed with cold PBS and stored at 4 °C until further processing.

### Cell viability assay

Cell viability was measured using the CCK-8 assay (Dojindo, Kyushu Island, Japan). At brief, BV2 microglial cells were seeded in 96-well plates, transient transfected with shRNA or pretreated with inhibitors, and then treated with various concentrations of pinocembrin under IH condition for 3, 6, 12, or 24 h. The cells were then treated with 10 μL/100 μL medium CCK-8 assay reagent and incubated for 2 h at 37 °C in a 5% CO_2_ incubator. Optical density (OD) was measured at 450 nm under a microplate spectrophotometer (Thermo Fisher Scientific, Camarillo, CA, USA).

### Analysis of mitochondrial ROS (mtROS)

Mitochondrial ROS (mtROS) level in living BV2 cells was detected by Mito SOX Red (Invitrogen) assay according to the manufacturer’s instructions. Briefly, the cells were seeded in 6-well plates with a density of 5 × 10^5^/ml with 3 parallel wells in each group. After various treatments, cells were stained with MitoSOX Red probe at a final concentration of 5 μM at 37 °C in the dark for 10 min. Then, cells were washed thoroughly with PBS, and the activity of mtROS was analyzed using FACSCalibur (BD Biosciences). Quantification was conducted by FlowJo software (Tree Star, San Carlos, CA).

### Mitochondrial membrane potential (MMP)

Changes in MMP were determined using a JC-1 mitochondrial membrane potential assay kit (Beyotime, China) according to the manufacturer’s instructions. All samples were observed under an inverted microscope (Olympus IX73, Japan) or estimated quantitation of fluorescence intensity using FACSCalibur (BD Biosciences). The loss of MMP was reflected by the ratio of aggregates (red fluorescence) to monomer (green fluorescence).

### Transmission electron microscopy (TEM)

BV2 cells were harvested in 1 mm^3^ and fixed in ice-cold 2.5% glutaraldehyde. Next, samples were dehydrated in ethanol (with 3% uranyl acetate), embedded in a mixture of epoxy resin and propylene oxide for 24 h. After a 70-nm-thick sample section staining with lead citrate, the mitochondrial morphology and mitophagosomes were detected under a transmission electron microscope (JEM 1011, Japan) by two blinded pathologists.

### Immunoprecipitation (IP)

Total protein lysate from BV-2 cells was immunoprecipitated with 1 μg of anti-LC3 antibody overnight at 4 °C. Protein A agarose beads (30 μl, Cell Signaling Technology) was added to the mixture and incubated at 4 °C for 4 h. The beads were collected by centrifugation at 12,000×*g* for 60 s at 4 °C and then washed 3 times by immunoprecipitation buffer. Protein complexes were resuspended in 5× loading buffer and boiled for 10 min. Then, the collected supernatant was used in western blotting.

### Statistics

All statistical analyses were conducted using SPSS 25.0 software and Prism 7.0 (GraphPad). Differences between two groups were analyzed by two-tailed unpaired Student’s *t* test. One-way analysis of variance (ANOVA) followed by Tukey’s post hoc test was used for multiple comparisons. In addition, escape latencies during the training of WMW were analyzed by two-way repeated measures ANOVA, while platform-crossing times were analyzed by ANOVA on ranks. Qualitative data were expressed as means ± standard error of the mean (SEM). Significance was considered when *p* < 0.05.

## Results

### Pinocembrin improves behavior and cognitive deficits in IH mice

To determine the effects of pinocembrin on spatial learning and memory of the IH mice, the MWM test was performed. The mice were trained to remember the location of the escape platform for 5 consecutive days, and the data showed that the latency was significantly increased for IH-treated mice as compared to the NA group (*p* < 0.05). The results also revealed the preservation of learning ability in mice treated with pinocembrin relative to the mice after exposures with IH (*p* < 0.05, Fig. 1b). Meanwhile, the memory activities were then tested after the platform had been removed. During the probe trail, the mice from the IH group spent less time in the target quadrant than those from the NA group (*p* < 0.01). Pinocembrin treatment obviously rescued the cognitive decline induced by IH (*p* < 0.05; Fig. 1c). Moreover, compared to the NA group, the frequency to cross the platform was decreased in the IH group. This decrease was reversed by pinocembrin treatment (*p* < 0.05; Fig. 1d). In addition, swim speed of the IH and pinocembrin treatment groups did not significantly change compared to the NA group, thereby suggesting no differences between the groups in terms of locomotor ability or coordination (Fig. 1e). Taken together, these results suggest that pinocembrin treatment can alleviate the cognitive decline induced by IH in mice.

### Pinocembrin ameliorates microglia-mediated neuroinflammation in IH mice

**Fig. 2:**
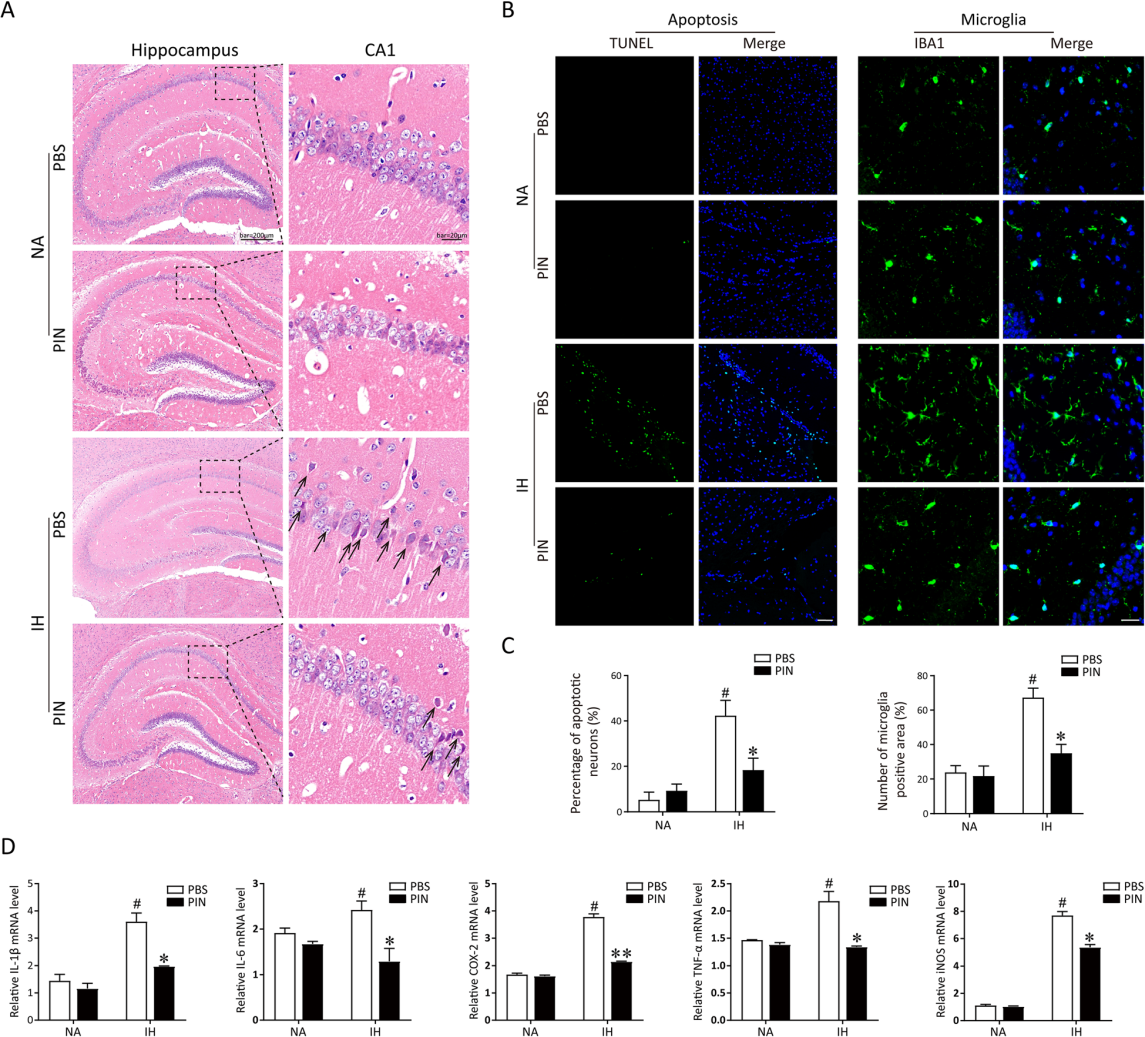
Pinocembrin treatment prevents IH-induced neuroinflammation via inhibiting microglia-mediated inflammation. **a** The typical HE staining was performed on the hippocampus of mice. IH exposure resulted in a neuronal degeneration in CA1 hippocampus (black arrow), which can be alleviated by pinocembrin treatment (40 mg/kg). **b** The neuronal apoptosis was detected using TUNEL staining. Representative immunofluorescent micrographs for TUNEL (green) staining from the hippocampus region of each group. Bar = 50 μm. Microglia were stained with ionized calcium-binding adapter molecule 1 (Iba1) antibody. IH exposure significantly increased the infiltration of microglia in hippocampus tissues, which can be alleviated by pinocembrin treatment. Bar = 20 μm. c Quantitative analyses of the number of apoptotic cells and microglia. **d** Pinocembrin could significantly decrease the secretion of IH-induced inflammatory cytokine (TNF-α, iNOS, COX-2, IL-6, and IL-1β) in the hippocampus of mice. The expressions of mRNAs were analyzed by qRT-PCR and normalized to GAPDH. ^#^*p* < 0.01 vs. the control group; *p < 0.05 vs. the IH group; **p < 0.01 vs. the IH group

To determine whether pinocembrin protects the neurons from IH-induced damage, the histological photographs for the examination of neuronal apoptosis from the different groups are shown in Fig. 2a. The neurons in CA1 regions of the hippocampus were arranged in order in the NA and PIN groups. IH treatment significantly reduced the number of neurons and resulted in a severe neuronal degeneration at the hippocampus, with nucleolus disappears and triangulated neuronal body (black arrow), while in the pinocembrin (40 mg/kg) group, neurons had enlarged soma and normal morphology compared with the IH group. We then used the TUNEL assay to assess cell apoptosis in experimental models of sleep apnea. TUNEL staining showed that few apoptotic neurons were detected in the hippocampus of mice in the NA and PIN groups and that higher numbers of TUNEL-positive neurons were found in the IH group (*p* < 0.01 vs. NA group). Administration of pinocembrin (40 mg/kg) significantly decreased the proportion of TUNEL-positive neurons in the hippocampus in IH-treated mice (*p* < 0.05; Fig. 2b, c).

Abnormally activated microglia are reported to be the main cells mediating neuroinflammation [11]. At the completion of 21-day IH treatment, the expression of Iba-1 was assessed in hippocampus tissues via immunofluorescent staining. Hippocampal tissue from IH group mice showed a great deal of Iba-1-positive cells relative to the NA group (*p* < 0.01). Compared with the IH group, treatment with 40 mg/kg pinocembrin significantly reduced the number of Iba-1-positive cells in the hippocampal tissue (*p* < 0.05; Fig. 2b, c), demonstrating that pinocembrin administration inhibited the infiltration and activation of microglia in the hippocampus after IH exposure. We then tested the effects of pinocembrin on the production of inflammatory mediators in hippocampi with qRT-PCR. The results showed that pinocembrin treatment visibly suppressed the production of IH-induced inflammatory cytokine (Fig. 2d). Taken together, these data reveal that pinocembrin treatment can ameliorate microglia-mediated neuroinflammation in IH mice.

### Pinocembrin inhibits NLRP3 inflammasome activation and enhances BNIP3-mediated mitophagy in hippocampi of IH mice

**Fig. 3:**
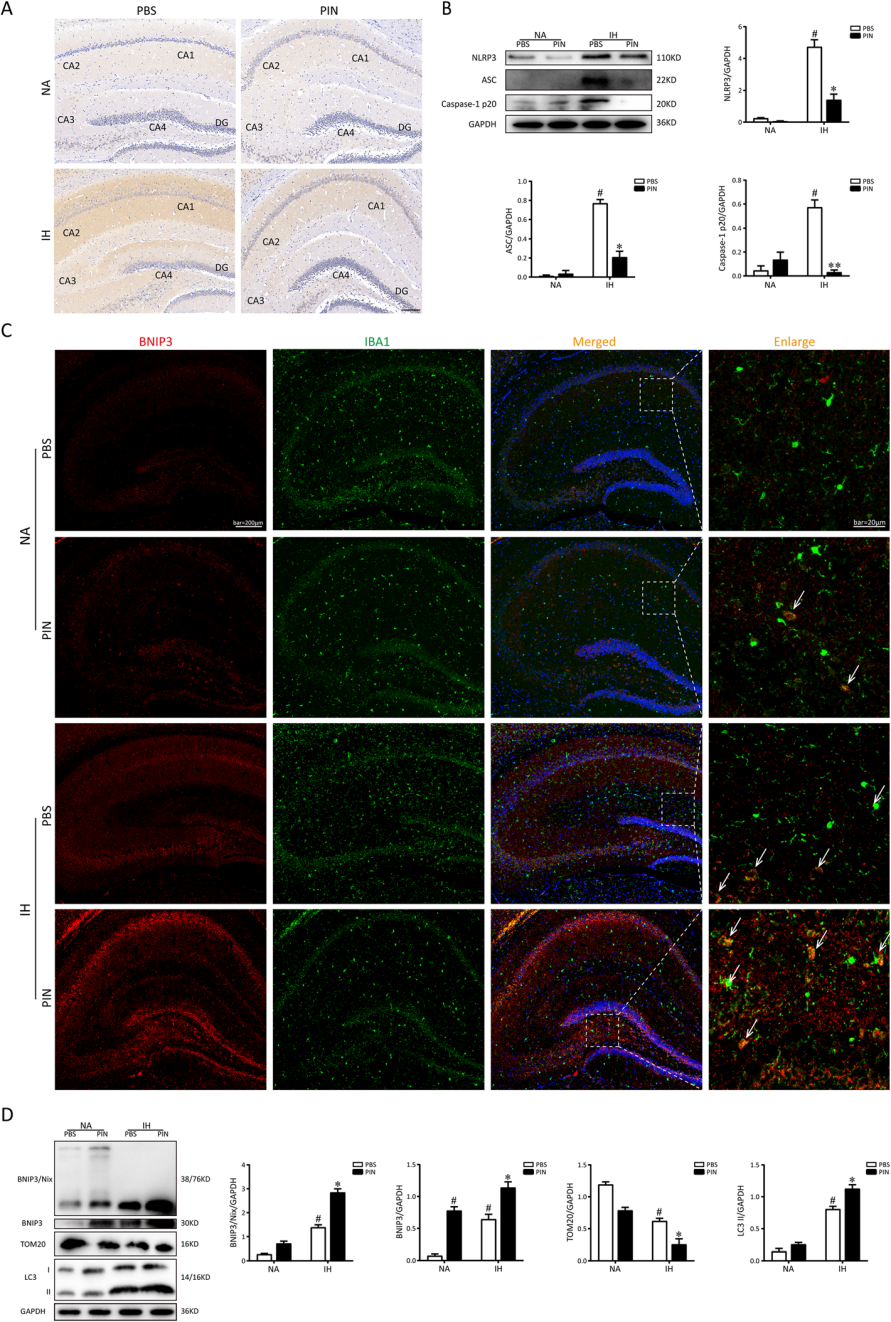
Pinocembrin treatment inhibits NLRP3 inflammation activation and enhances BNIP3-mediated mitophagy following IH exposures in mice. **a** The immunohistochemistry of NLRP3 in the hippocampus after exposure to IH treatment with or without pinocembrin. Bar = 100 μm. Western blots of NLRP3, caspase-1 p20, and ASC in the hippocampi from mice exposed to NA or IH, treating with vehicle or pinocembrin, respectively. **b** Western blots of NLRP3, caspase-1 p20, and ASC in the hippocampi from mice exposed to NA or IH, treating with vehicle or pinocembrin, respectively. **c** Representative pictures of double staining with BNIP3/IBA1 (white arrow) in hippocampi of mice obtained by a fluorescence microscope. Pinocembrin administration further increased the level of BNIP3 following IH exposures of hippocampi in mice. **d** Mice were exposed to NA or IH for 21 days and hippocampi were collected. Typical western blotting images of BNIP3/NIX, BNIP3, TOM20, and LC3 proteins of hippocampi in mice. ^#^*p* < 0.01 vs. the control group; *p < 0.05 vs. the IH group; **p < 0.01 vs. the IH group

We determined the effects of pinocembrin on the expression of NLRP3, ASC, and cleaved caspase-1 in the hippocampus of the mice after exposure with IH by immunohistochemistry and western blotting, respectively. Compared with the NA group, IH exposure led a marked increase in the expression of NLRP3 in the hippocampus. Pinocembrin treatment can observably reverse these changes (Fig. 3a). Similar results were obtained from western blot analysis. As shown in Fig. 3b, IH mice had increased protein levels of NLRP3, cleaved caspase-1, and ASC in the hippocampus compared to the NA group. The levels of those proteins were markedly reduced by pinocembrin treatment after exposing to IH. These results indicate that the NLRP3 inflammasome activation may contribute to IH-induced hippocampal inflammation, which could inhibit pinocembrin.

Next, to detect the BNIP3-related mitophagic activity in the hippocampus of mice, immunofluorescence stain and western blotting were performed. As illustrated in Fig. 3c, typical immunofluorescence images of doubling staining using BNIP3/IBA-1 revealed that administration of 40 mg/kg pinocembrin can further increase BNIP3 protein expression after IH exposure. Furthermore, western blot analysis of proteins from the hippocampus of mice showed that the expressions of BNIP3/NIX, BNIP3, and LC3 were low in the NA group. In comparison, the expressions of BNIP3/NIX, BNIP3, and LC3 were significantly higher in the IH and PIN groups. The levels of TOM20 protein was in a reverse tendency, demonstrating the activation of BNIP3-mediated mitochondrial autophagy. Additionally, administration of 40 mg/kg pinocembrin companied with IH exposure further activated BNIP3-relative mitophagy (Fig. 3d). Therefore, these findings demonstrate that pinocembrin can further activate BNIP3-mediated mitophagy in the hippocampus of IH mice.

### Pinocembrin exerts positive effects on IH-treated microglia in vitro

**Fig. 4:**
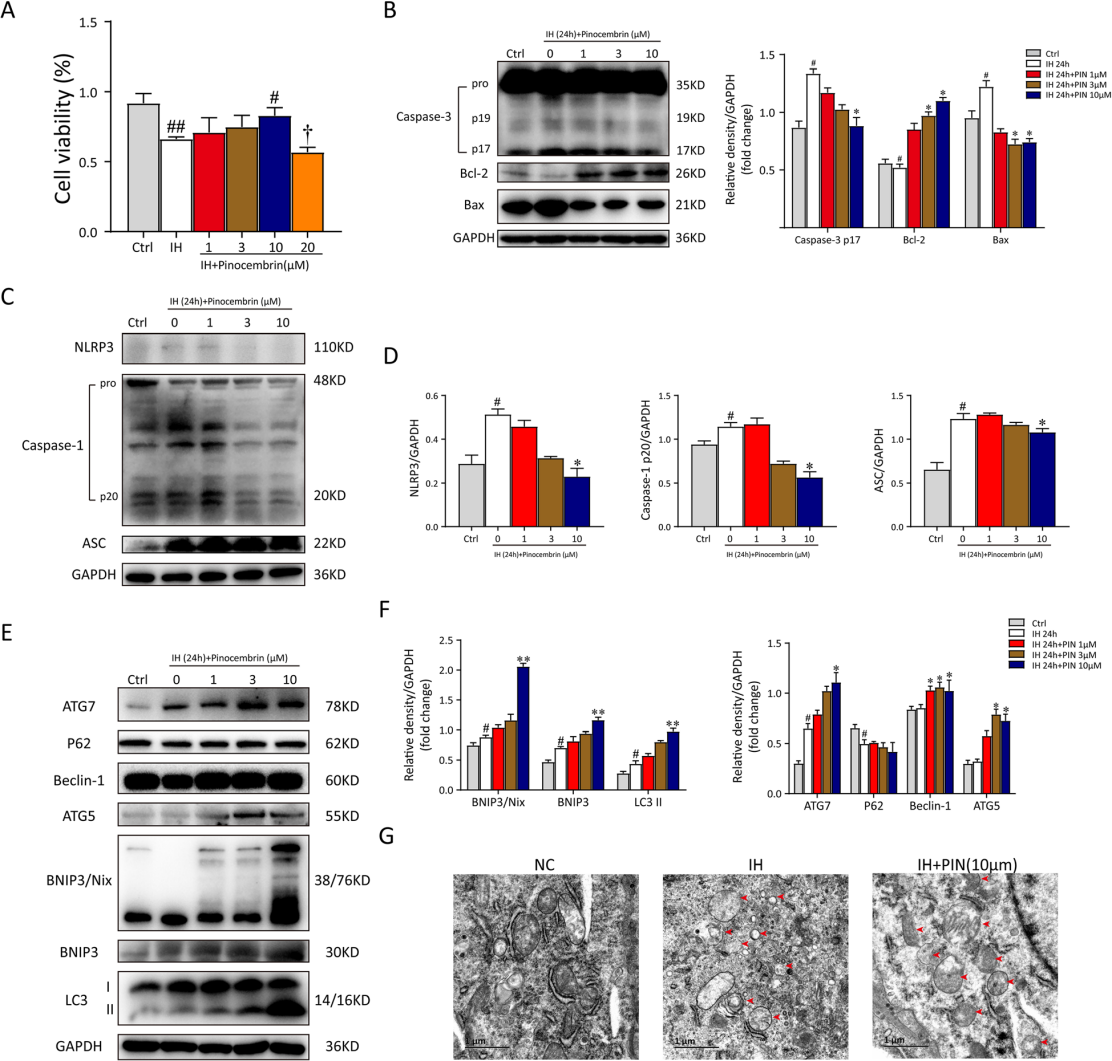
Pinocembrin shows protective effects on IH-treated microglial cells and enhances BNIP3-mediated mitophagy. **a** Microglial cells were incubated with pinocembrin at different concentrations (0, 1, 3, 10, and 20 μM) under intermittent hypoxia (IH) condition for 24 h. Then, the viability of cells was measured by Cell Counting Kit-8 assay kit. **b** Pinocembrin exposure significantly reduced IH-induced cell apoptosis, and protein expressions of caspase-3, Bcl-2, and Bax were detected by immunoblotting. Pinocembrin at the concentration of 10 μM for BV2 cells with IH exposure showed optimal results in all. GAPDH worked as the loading control. **c** Western blot analysis of NLRP3, caspase-1, and ASC expression in microglial cells treated with different concentrations of pinocembrin (0, 1, 3, and 10 μM). **d** The relative optical density values of mentioned proteins to GAPDH were analyzed and demonstrated as statistical graphs, respectively. **e** Western blot was used for autophagic and mitophagic activation determination in groups with different concentrations of pinocembrin (0, 1, 3, and 10 μM). GAPDH worked as the internal control for whole cellular protein extraction. **f** The densitometric analysis of ATG7, P62, Beclin-1, ATG5, BNIP3/Nix, BNIP3, and LC3 expression. Pinocembrin exposure further increased autophagic and mitophagic protein expression. **g** Electron micrographs showed mitochondrial damages and mitophagosomes (red arrows) in microglial cells. Data represented as mean ± SEM from 3 independent experiments. ^#^*p* < 0.05 vs. the Ctrl group; ^##^*p* < 0.01 vs. the Ctrl group; *p < 0.05 vs. the IH group; **p < 0.01 vs. the IH group; ^†^*p* < 0.05 vs. the IH + 10 μM group

To establish an in vitro IH-activated microglial model, we incubated BV2 cells in an IH environment, as previously described [14]. Cell viability was significantly lower in the IH-treated BV2 cells than in the NA cells at 24 h. Therefore, long-term IH exposure can be detrimental to these cells. We investigated the therapeutic and cytotoxic effects of pinocembrin on IH-treated BV2 cells. The cells were pretreated with pinocembrin at a concentration of 1, 3, 10, or 20 μM for 1 h before exposure with IH for 24 h, and then their viability was evaluated with a CCK-8 assay. As shown in Fig. 4a, there was a significant increase in the cell survival rate in the IH + 10 μM group compared with that in the IH group. We also found that 20 μM pinocembrin elicited a robust inhibitory effect on cell viability, indicating that 10 μM is the optimum concentration of pinocembrin for BV2 cells. Immunoblotting analysis of whole-cell lysates showed that the expression of cleaved caspase-3 and Bax was significantly elevated and the Bcl-2 protein level was reduced (Fig. 4b). It is generally believed that inflammatory responses are implicated in cellular oxidative stress and the activation of the NLRP3 inflammasome, which are vital for the fates of cells [16, 25]. Immunoblotting analysis showed that pinocembrin exerted a dose-dependent inhibitory effect on the activation of NLRP3 inflammation (1, 3, or 10 μM), evident as reduced levels of NLRP3, cleaved caspase-1, and ASC (an apoptosis-associated speck-like protein containing a caspase-recruitment domain) proteins (Fig. 4c, d). These data indicate that pinocembrin alleviates IH-induced cytotoxicity by preventing the accumulation of NLRP3 inflammasomes and by decreasing cell apoptosis.

We then assessed autophagy and mitophagy in the microglia, two of the major mechanisms in the CNS that keep inflammation in check [7]. To examine the relationship between the pinocembrin-mediated neuroprotective effect and mitophagy, we examined the activities of autophagy and mitophagy with an immunoblotting analysis. An immunoblotting analysis of ATG7, P62, Beclin-1, ATG5, BNIP3/Nix, BNIP3, and LC3-II revealed the dose-dependent activation of autophagy and mitophagy by treating with pinocembrin (Fig. 4e, f). Further examination by using TEM confirmed mitochondrial damages and mitophagosome formation (red arrow) in BV2 cells, which were rarely observed in microglial cells from the NA group. IH exposure exhibited profound effects on mitochondrial structure, including the increase in vacuoles in the mitochondrial matrix, mitochondrial fragmentation and swelling, and loss of cristae. In addition, compared with the IH group, there were plenty of autophagosome and mitophagosome formations (red arrow) observed in the cytoplasm of the microglia in the IH + PIN group (Fig. 4g). Taken together, these results demonstrate that Bnip3-related mitophagy is involved in the protective effect of pinocembrin.

### Pinocembrin suppresses IH-induced microglial cell damage via BNIP3-dependent mitophagy

**Fig. 5:**
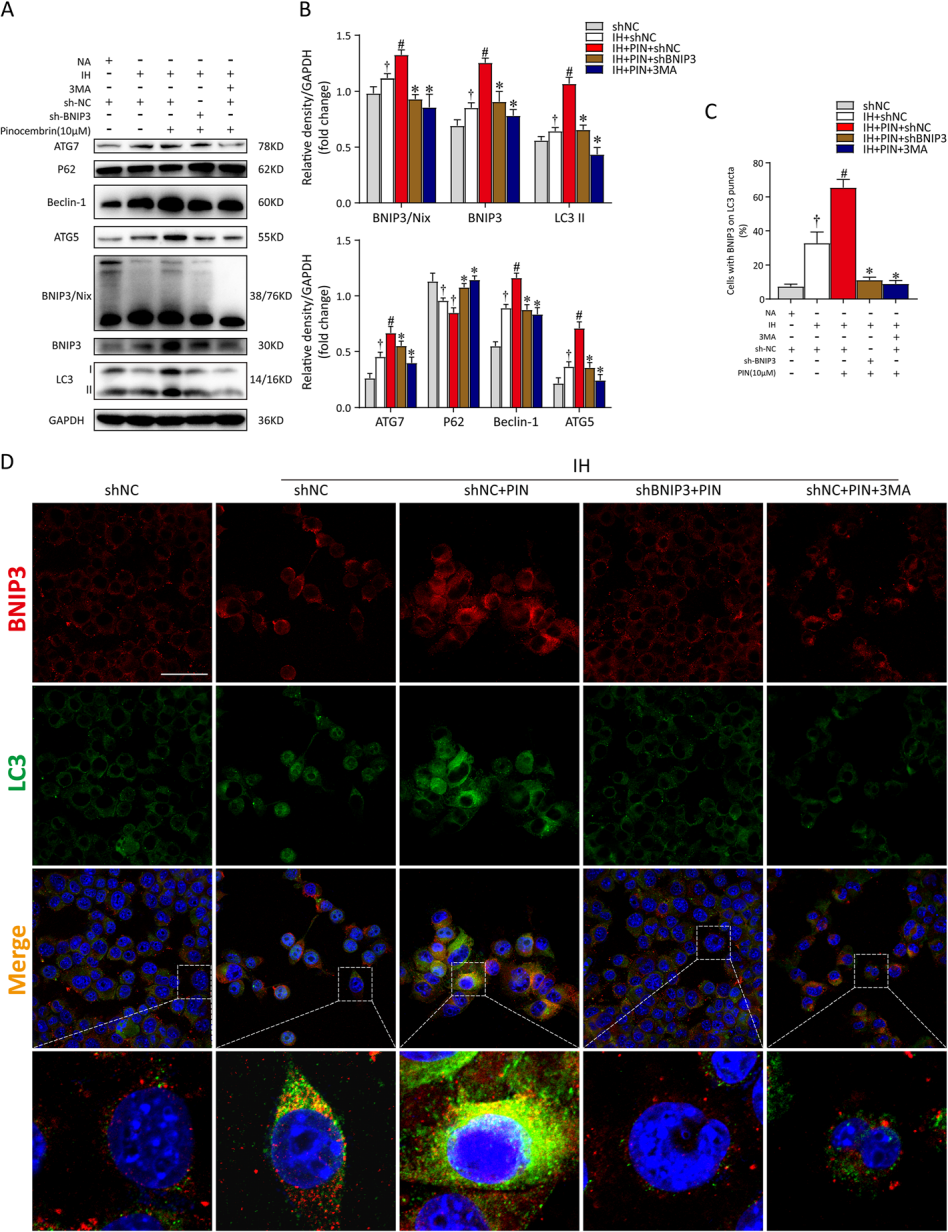
Pinocembrin enhances mitochondria-mediated autophagy through the upregulation of BNIP3. sh-BNIP3 plasmid-transfected BV2 cells were pretreated with 3MA (autophagy inhibitor, 5 mM) for 6 h or pinocembrin (10 μM) for 1 h before treatment with intermittent hypoxia (IH). **a** Representative western immunoblots for autophagic and mitophagic markers (ATG7, P62, Beclin-1, ATG5, BNIP3/Nix, BNIP3, and LC3-II) in BV2 cells with different treatments. **b** Bar graphs showing corresponding quantitative data of protein expressions. **c** Bar graph summarizing the double-labeling data (BNIP3 and LC3) obtained from BV2 cells. Staining cells were counted a total cell sampling of 500–1000 randomly chosen from 3 to 5 fields in each experiment. **d** Confocal microscopy view of the transfected BV2 cells with or without pinocembrin or 3MA respectively. DAPI was used to stain nuclei. Bar = 50 μm. Data are representative of at least 3 independent experiments and presented as mean ± SEM in all assays. ^†^*p* < 0.05 vs. the NA + sh-NC group; ^#^*p* < 0.05 vs. the IH + sh-NC group; *p < 0.05 vs. the IH + sh-NC + PIN group

To further investigate the role of BNIP3 in the neuroprotective action of pinocembrin, we transfected cells with shRNA or incubated with 3-MA (an inhibitor of autophagy, 5 mM) before pinocembrin treatment (10 μM). We first used western blotting to evaluate the changes in autophagic and mitophagic activities. Our results indicated that silencing BNIP3 or pretreatment with 3MA neutralized the effect of pinocembrin and reduced the levels of both autophagy and mitophagy in IH-treated microglial cells (Fig. 5a, b). We then used double immunofluorescent staining for the autophagic markers LC3 (green) and BNIP3 (red) in microglial cells to clarify the changes in BNIP3-mediated mitophagy. The stain intensity of LC3 staining and the numbers of cells expressing BNIP3 were clearly increased in the IH microglia treated with pinocembrin. However, after transfection with shBNIP3 or pretreatment with 3MA, pinocembrin no longer enhanced mitophagy after IH exposure. As shown in Fig. 5c and d, a number of puncta (yellow) showed faint immunofluorescence in the shBNIP3 and 3MA groups. Moreover, the co-localization of LC3 and BNIP3 demonstrated the declined mitophagosome formation after BNIP3 inhibition.

**Fig. 6:**
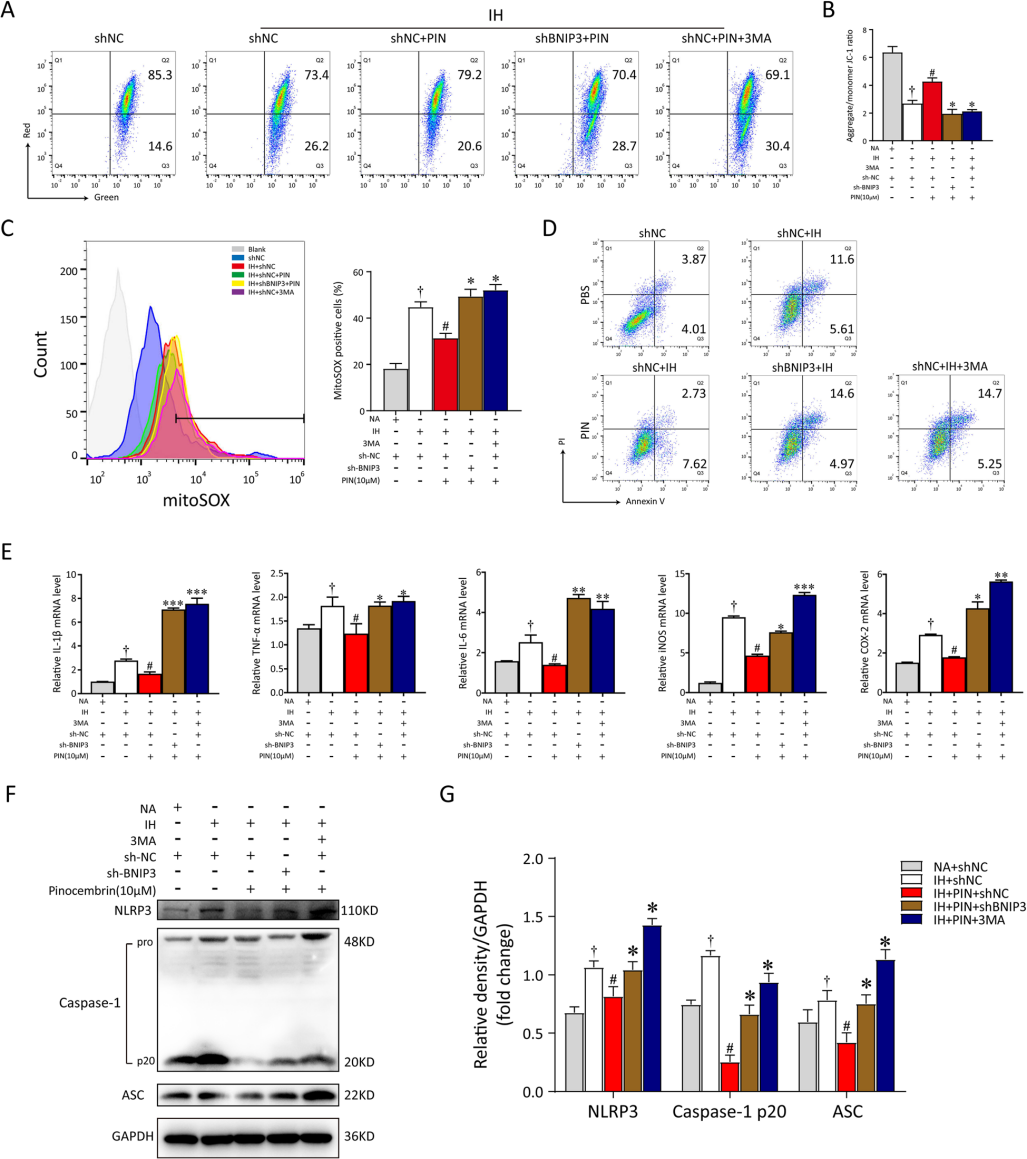
Pinocembrin alleviates IH-induced mitochondria damage and cell inflammation via BNIP3-dependent mitophagy. **a** The changes of mitochondrial membrane potential (MMP) in BV2 cells with different treatments were analyzed quantitation of fluorescence intensity through flow cytometry. **b** Quantitative analysis of MMP was represented as the ratio red to green fluorescence. **c** The levels of cellular mitochondrial ROS (mtROS) were determined by mitoSOX^TM^ red mitochondrial superoxide indicator using flow cytometry. The quantitation of fluorescence intensity suggested BNIP3 deficiency markedly increased mtROS production, and induced mitochondria damage against the protective effect from pinocembrin. **d** sh-NC or sh-BNIP3-transfected BV2 cells were exposed to IH for 24 h, and then cells were double-stained with annexin V/PI. The proportion of apoptotic cells were detected by flow cytometry. **e** mRNA levels for different inflammatory cytokine genes (TNF-α, iNOS, COX-2, IL-6, and IL-1β) in shNC, shBNIP3, PIN, and/or 3MA groups. **f**, **g** Total cell lysates prepared from treated microglia were separated on SDS-PAGE gel and detected by western blot analysis. Data are representative of at least 3 independent experiments and presented as mean ± SEM in all assays. ^†^*p* < 0.05 vs. the NA + sh-NC group; ^#^*p* < 0.05 vs. the IH + sh-NC group; *p < 0.05 vs. the IH + sh-NC + PIN group; **p < 0.01 vs. the IH + sh-NC + PIN group; ***p < 0.005 vs. the IH + sh-NC + PIN group

To examine the changes in mitochondrial function more closely, the levels of MMP and mtROS production were also assessed. According to our in vitro data, pinocembrin treatment markedly restored the disrupted MMP (red to green ratio, *p* < 0.05, 2.80 ± 0.89% vs. 3.84 ± 1.11%) and attenuated the release of mtROS (*p* < 0.05, 44.2 ± 1.88% vs. 31.30 ± 2.25%). After transfection with shRNA or stimulation with 3MA, the positive effects of pinocembrin on IH microglia were absolutely abrogated. The loss of MMP was dramatically enhanced by BNIP3 deletion or 3MA treatment (2.45 ± 1.34%; 2.27 ± 2.88%), and mtROS were greatly upregulated (49.30 ± 1.79%; 52.03 ± 3.16%), as shown in Fig. 6a–c. The inhibition of BNIP3-dependent mitophagy also increased the proportion of apoptotic cells, despite pinocembrin treatment (12.03 ± 3.01%), as shown in Fig. 6d. In summary, these results show that pinocembrin maintains mitochondrial homeostasis and neutralizes excessive oxidative stress via Bnip3-dependent mitophagy. We then investigated whether BNIP3-dependent mitophagy is implicated in the inhibitory effect of pinocembrin on the NLRP3-mediated inflammatory response. As shown in Fig. 6e, the relative expression of mRNAs for TNF-α, iNOS, COX-2, IL-6, and IL-1β increased distinctly in the shBNIP3- or 3MA-treated BV2 cells, despite treatment with pinocembrin. Furthermore, immunoblotting analysis confirmed the inhibition of BNIP3-dependent mitophagy increased NLRP3 inflammasome activation, which abolished the therapeutic effect of pinocembrin on IH microglia (Fig. 6f, g). Taken together, these results indicate that pinocembrin exerts a protective effect in the context of IH by decreasing mtROS production and NLRP3 inflammasome formation, which relied on the activation of BNIP3-dependent mitophagy.

### Pinocembrin activates Bnip3-dependent mitophagy via the JNK-ERK signaling pathway

**Fig. 7:**
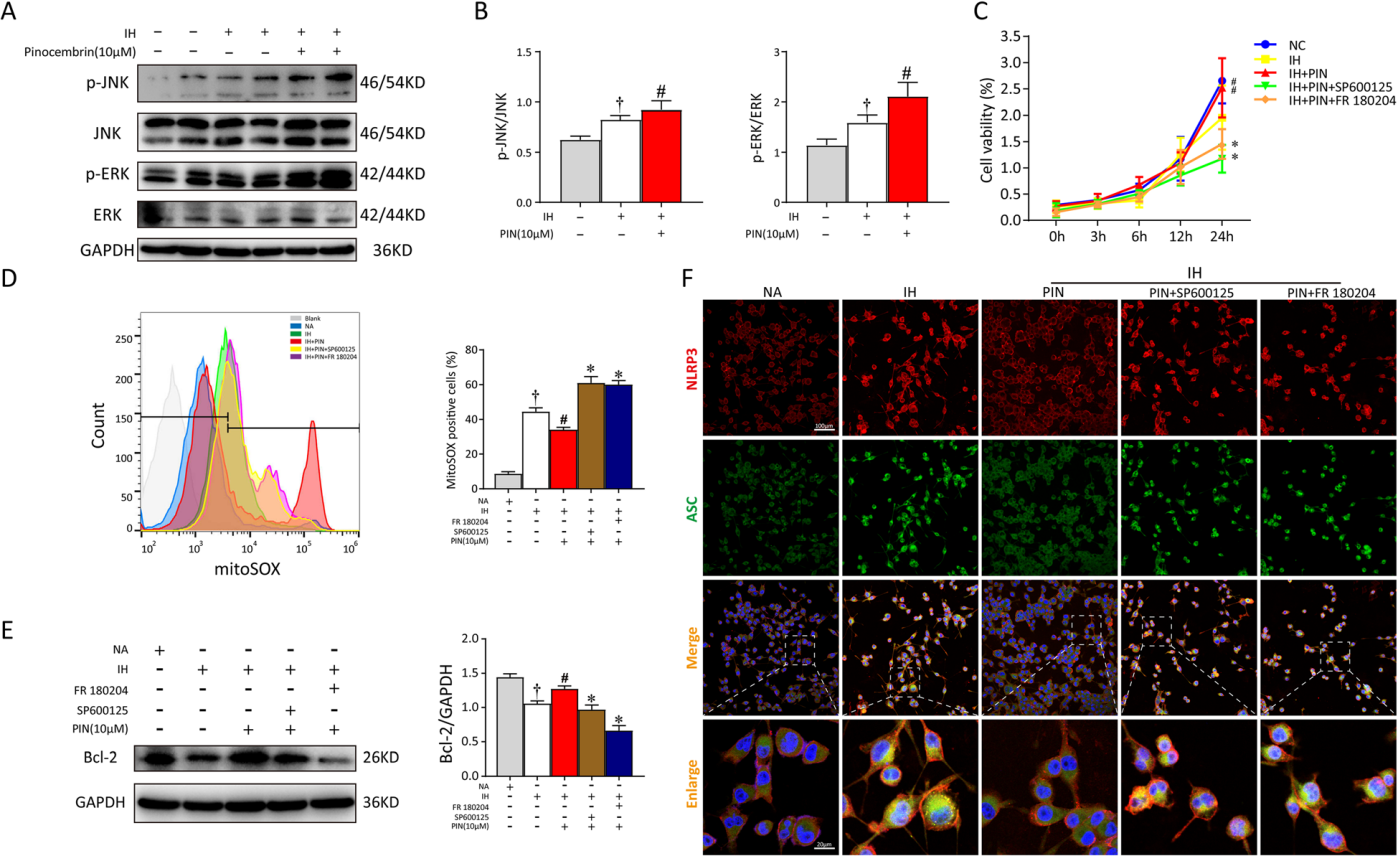
JNK-ERK AMPK pathway was involved in the protective effect of pinocembrin which activated BNIP3-mediated mitophagy. BV2 cells were pretreated with SP600125 (JNK inhibitor, 10 μM) or FR 108204 (ERK inhibitor, 10 μM) for 30 min before the pinocembrin administration. Pinocembrin was added to the culture medium for 1 h before exposing to NA (normal air) or IH (intermittent hypoxia). **a** Total proteins from the microglia were immediately separated by SDS-PAGE gel and analyzed by western blot. Blots were probed with specific primary antibodies as indicated. **b** Quantitation of pinocembrin-activated ERK1/2 and JNK MAPK pathways. Depicted is the weighted average density of bands (p-JNK and p-ERK) normalized to JNK or ERK at 24-h exposure with NA or IH, respectively. **c** Proliferation of BV2 cells in different groups was analyzed by CCK-8 assay. **d** Flow cytometric analysis of microglia for mtROS with MitoSOX. **e** Western analysis showed pinocembrin induced Bcl-2 expression under IH exposure, but reduced by JNK or ERK inhibitor. GAPDH was shown as a loading control. **f** Representative images of NLRP3 (red) and ASC (green) by immunofluorescent staining of BV2 cells. Data are representative of at least 3 independent experiments and presented as mean ± SEM. ^†^*p* < 0.05 vs. the NA group; ^#^*p* < 0.05 vs. the IH group; *p < 0.05 vs. the IH + PIN group

It has been reported that BNIP3-induced mitophagy occurs via JNK and ERK MAPK activation [7, 19]. To further investigate the underlying molecular mechanisms of pinocembrin-mediated neuroprotection, the effects of pinocembrin on IH-elicited JNK and ERK phosphorylation were tested. The phosphorylation of JNK and ERK was significantly increased in the IH + pinocembrin group, and in particular, the level of p-ERK was approximately 1.5-fold higher than that in the IH group (Fig. 7a, b). We then added SP600125 (JNK inhibitor, 10 μM) or FR 108204 (ERK inhibitor, 10 μM) to detect the changes in cell viability, cell apoptosis, and mtROS-NLRP3 signaling pathway when the activity of JNK or ERK was inhibited, respectively. As shown in Fig. 7c, pinocembrin alone promoted the viabilities of IH microglia as compared to the IH group. The cell viabilities determined by CCK-8 assay of the inhibitor groups were obviously lower than those of pinocembrin-treated cells without SP600125 or FR 108204. After incubation with MitoSOX Red probe, the activity of mtROS in microglia was detected with flow cytometry. The levels of mtROS decreased greatly in cells treated with pinocembrin (24.23 ± 2.01%), whereas nearly 2-fold the increased level of oxidative stress was detected when the pinocembrin group was pretreated with inhibitor (SP600125, 60.17 ± 2.01%; FR 108204, 58.67 ± 1.77%; Fig. 7d). The rate of cell apoptosis was determined with immunoblotting. As shown in Fig. 7e, the dephosphorylation of p-JNK or p-ERK significantly inhibited the expression of Bcl-2 (an anti-apoptotic protein), suggesting that the inhibition of JNK and ERK activity exacerbates the detrimental actions of IH. Further double staining of NLRP3 (red) and ASC (green) revealed a marked increase in inflammasome formation in IH microglia treated with both inhibitors (Fig. 7f).

**Fig. 8:**
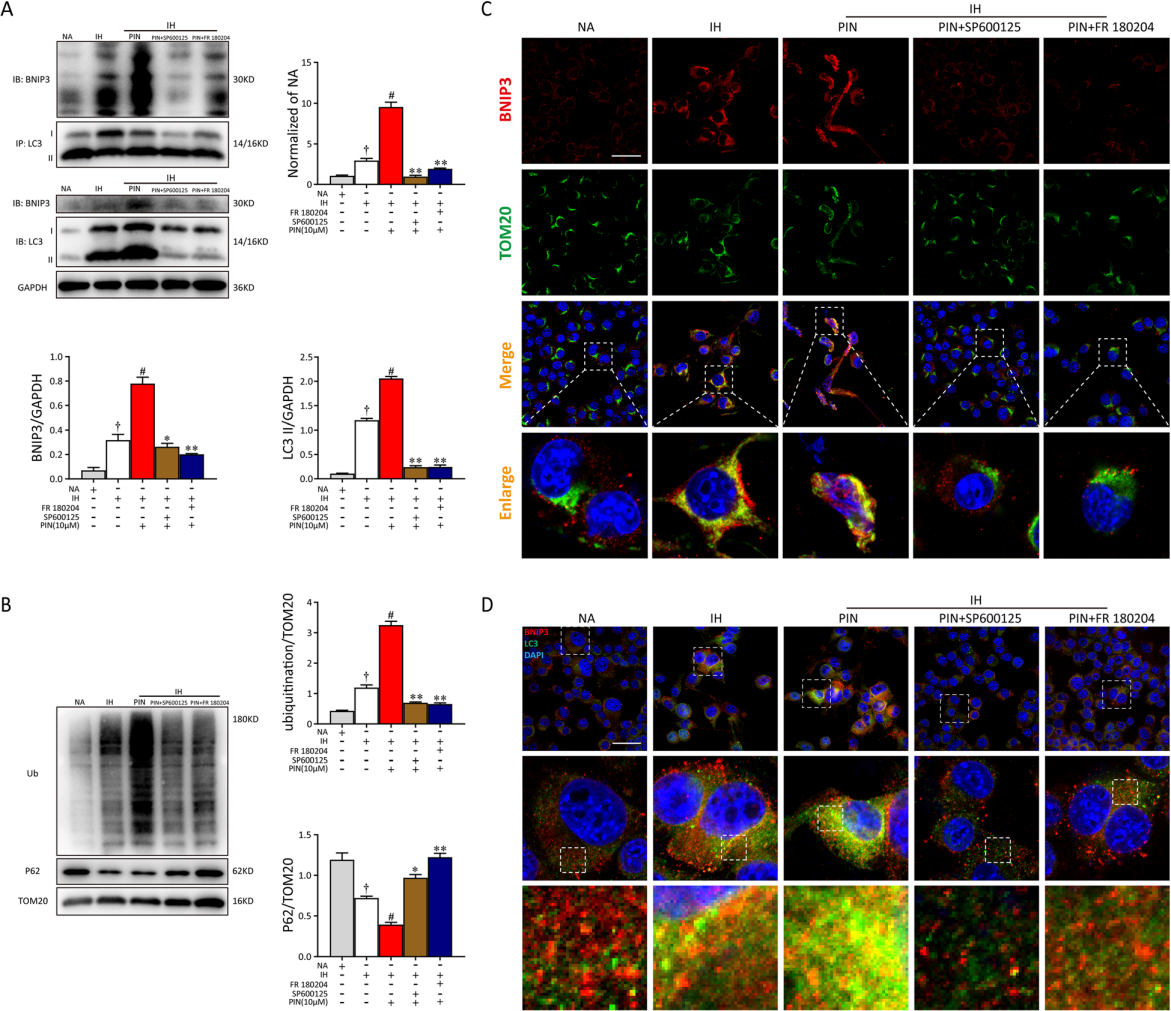
Pinocembrin-induced mitochondrial autophagy is mediated by the JNK/ERK/BNIP3/LC3 pathway. **a** Whole-cell proteins from BV-2 cells were harvested immediately after different treatments. Immunoprecipitation was performed by an anti-LC3 antibody and was immunoblotted for BNIP3 and LC3. **b** Representative western blotting images of ubiquitin and P62 of BV2 cell mitochondrial fraction. **c** Microglia were double-stained with BNIP3 and TOM20 (MitoTracker). Representative micrographs for BNIP3 (red), TOM20 (green), DAPI (blue), and merged pictures (yellow) from each group were captured by a confocal microscope. Bar = 50 μm. **d** The immunocytochemistry of BV2 microglial cells was conducted with anti-BNIP3 and anti-LC3 antibodies. Confocal microscopy revealed that the SP600125 and FR 108204 groups had few positive staining of autophagosome marker LC3 compared with the pinocembrin-only group, as well as less co-localizing with BNIP3. Bar = 50 μm. Data are representative of at least 3 independent experiments and presented as mean ± SEM. ^†^*p* < 0.05 vs. the NA group; ^#^*p* < 0.05 vs. the IH group; *p < 0.05 vs. the IH + PIN group; **p < 0.01 vs. the IH + PIN group

We then explored whether the phosphorylation of JNK or ERK was the upstream of BNIP3-dependent mitophagy in the pinocembrin-treated microglia. As shown in Fig. 8a, levels of BNIP3 and the LC3 complex increased upon pinocembrin pretreatment in BV2 cells. However, the interaction between BNIP3 and LC3 was decreased dramatically when microglia were co-incubated with inhibitors, indicating the inhibition of BNIP3-dependent mitophagy. To further confirm the role of p-JNK and p-ERK in pinocembrin-induced mitophagy, we performed western blotting using the lysates from mitochondrial fraction of BV2 cells. In contrast to the IH + pinocembrin group, inhibitors significantly abolished mitochondrial co-localization with P62 and mitochondrial ubiquitination in pinocembrin-treated microglial cells (Fig. 8b). Moreover, as shown in Fig. 8c, the BV2 cells showed further increases in BNIP3 (red) and TOM20 (mitochondrial-related protein, green) protein expression and their co-localization in response to pinocembrin exposure. Pretreatment with inhibitors prominently reversed these responses in pinocembrin-treated BV2 cells. Further immunofluorescent staining of LC3 (green) and BNIP3 (red) also showed that both SP600125 and FR 108204 suppressed the activation of BNIP3-dependent mitophagy, indicating that the level of BNIP3-dependent mitophagy was regulated by the activation of JNK and ERK (Fig. 8d). Taken together, these data demonstrate that the JNK-ERK signaling pathway is involved in the function of pinocembrin in maintaining mitochondrial homeostasis, inhibiting inflammation, and protecting microglial cell apoptosis during IH exposure via BNIP3-dependent mitophagy (Figure S1).

## Discussion

The present study demonstrated that the administration of pinocembrin can efficiently improve cognitive deficits and ameliorate brain injury in IH mice, evidenced by restoring microglial mitophagy and reducing neuronal apoptosis and hippocampal inflammation. This study also highlights a new signaling mechanism by which pinocembrin exerts its neuroprotective effects during IH via enhancing BNIP3-dependent mitophagy. Although several previous studies have proven the therapeutic action of pinocembrin during ICH [10, 12], few studies have focused on its neuroprotective effects during IH caused by OSA. To our knowledge, our data show for the first time that the pharmacological mechanism of pinocembrin involves the regulation of mitophagy and subsequently the inhibition of cell apoptosis and NLRP3-mediated inflammation during IH both in vivo and in vitro. Additionally, we suggest that BNIP3 is a potential target for the control of excessive mtROS production or NLRP3 inflammasome accumulation in brain injury and that pinocembrin has therapeutic potential for OSA-associated neuroinflammation.

One of the pathophysiological hallmarks of OSA is the presence of neuroinflammation, which is closely associated with the microglia [2]. It has also been known that oxidative stress and inflammation have negative mechanistic effects on the microglia, leading to their apoptosis. Damaged microglia release a large amount of pro-inflammatory cytokines, which further promote the cascade reactions of inflammation [26]. A previous study showed that the suppression of cell apoptosis can alleviate microglia-mediated inflammation [22]. Generally, the moderate activation of inflammation favors neural protection, but can lead to neurotoxicity when excessive [26]. Moreover, increasing evidence indicates that the NLRP3-associated inflammatory response plays an important role in the development of neuroinflammation [16]. Therefore, protecting microglia against inflammation-induced apoptosis is a strategy for alleviating neuroinflammation. In the last few years, increasing studies have shown that mitophagy, a mitochondrium-specific process of “self-digestion,” is an intrinsic anti-inflammatory mechanism, which initiates the clearance of dysfunctional mitochondria and restricts excessive inflammatory cytokine production, ultimately inhibiting nerve inflammation [27]. Therefore, our observations in the present study show that a potential mitophagy agonist (pinocembrin), which attenuates IH-induced mtROS production and the NLRP3-related inflammatory response by upregulating of BNIP3 expression, may offer a novel therapeutic strategy for neuroinflammation-related diseases.

Previous studies have demonstrated that pinocembrin has anti-inflammatory properties, which are absorbed and metabolized well after its oral administration [9]. In vitro experiments have also shown that pinocembrin inhibits LPS-induced inflammatory cytokine secretion in RAW264.7 cells [23]. Zhao et al. suggested that pinocembrin mitigates cell death by increasing autophagic activity, thus protecting the brain from ischemia/reperfusion injury [10]. In the present study, we examined the positive role of pinocembrin in IH-mediated brain tissue impairment to determine the ameliorative potential of pinocembrin in this context. Our findings indicate that the exposure of microglial cells to IH markedly reduced MMP and increased NLRP3-related inflammation, which were accompanied by the low-level upregulation of the mitophagy markers, BNIP3 and BNIP3/Nix. However, by using the pinocembrin, both autophagy and BNIP3-dependent mitophagy increased dramatically, inhibiting the mtROS-NLRP3 axis and inflammatory cytokine production. Therefore, our data show that pinocembrin inhibits mitochondrial damage and cell apoptosis by functionally activating BNIP3. Although pinocembrin provides a therapeutic advantage in neuroinflammatory states by upregulating BNIP3-dependent mitophagy in microglia, the exact role of autophagy in this process remains an enigma. In contrast, some investigators have reported that pinocembrin suppresses cell apoptosis by inhibiting autophagy [28, 29], which may be attributable to differences in the cell types examined.

Previous studies indicated that BNIP3 induces autophagy, accompanied by the loss of MMP and cell death [18, 30, 31]. Nevertheless, our results show that BNIP3 acts as a protective regulator during IH exposure, inducing mitophagosome formation and removing unhealthy mitochondria from microglial cells. Besides, BNIP3 deficiency results in impaired mitophagy which further accelerates cleaved caspase-1 release via NLRP3 inflammasome activation, indicating that BNIP3-dependent mitophagy acts as a pivotal brake to restrain mtROS-activated inflammatory cytokine secretion. We also demonstrate that BNIP3 controls the quality of the mitochondria by regulating MMP and mtROS production during IH [32]. Consistent with our data, Dhingra and his colleagues indicated that the BNIP3-mediated mitophagic pathway opposes the cell apoptotic pathway (involving caspase-3) because the autophagy of damaged mitochondria counteracts cell death signaling [33]. Moriyama et al. [19] showed that BNIP3-induced autophagy, triggered by the activation of JNK and ERK MAPK, played a crucial role in protecting mitochondrial homeostasis against irradiation-induced cell death. Recently, accumulating evidence has shown the protective effects of BNIP3 induction under hypoxic conditions, which is attributed to the upregulation of hypoxia-inducible factor 1 (HIF-1) [17]. However, the role of BNIP3 in hypoxia-induced cell death is controversial. Rikka et al. proved that BNIP3 contributes to the impairment of the mitochondrial respiratory chain, whereas autophagy is induced in response to Bnip3 and BNIP3L, whether or not the intrinsic (mitochondrial) cell death pathway is activated [31, 33]. As a consequence, whether BNIP3 activates mitophagy and then participates in the alleviation of cell death requires clarification. Interestingly, it has been documented that hypoxia-induced mitophagy is mediated by various factors in different cell types [32]. Although we have found that BNIP3-dependent mitophagy is associated with the neuroprotective effects of pinocembrin, it is still vague whether it plays a dominant role in the inhibition of IH-induced neuroinflammation. The relationships between pinocembrin and various mitophagy receptors have not been described in this study and warrant further research.

## Conclusions

In conclusion, pinocembrin significantly reverses the IH-induced cognitive decline and neuroinflammation in IH mice. In particular, our observations show that pinocembrin protects microglial cells against IH-induced cytotoxicity by activating BNIP3-dependent mitophagy through the JNK-ERK signaling pathway. Taken together, our preliminary experiments suggest that mtROS and NLRP3-mediated inflammation play key roles in the pathogenic processes driven by IH-induced microglia in OSA patients, which can be offset by pinocembrin-enhanced mitochondrial autophagy. Overall, these findings demonstrate that pinocembrin is a potential therapeutic agent for OSA-associated neuroinflammation.

## Supplementary Information

**Additional file 1:** **Table S1.** qRT-PCR primer sequences used in this study.

**Additional file 2:** **Figure S1.** Pinocembrin protects against IH-induced neuroinflammation by upregulation of BNIP3-dependent mitophagy through JNK and ERK MAPK signalling pathways.

## Data Availability

All data generated and/or analyzed during this study are included in this article.

## Abbreviations

BNIP3: BCL2-interacting protein 3
CNS: Central nervous system
DMSO: Dimethylsulfoxide
ERK: Extracellular regulated kinase
FBS: Fetal bovine serum
FITC: Fluorescein isothiocyanate
H&E: Hematoxylin and eosin
IP: Immunoprecipitation
IH: Intermittent hypoxia
IL-1β: Interleukin-1β
JNK: c-Jun N-terminal kinase
LPS: Lipopolysaccharide
mtROS: Mitochondrial ROS
MWM: Morris water maze
NF-κB: Nuclear factor-κB
NLRP3: Nucleotide-binding domain-like receptor protein 3
OSA: Obstructive sleep apnea
OD: Optical density
PFA: Paraformaldehyde
PI3K: Phosphoinositide 3-kinase
PVDF: Polyvinylidene difluoride
PI: Propidine iodide
ROS: Reactive oxygen species
SEM: Standard error of the mean
TUNEL: Terminal deoxynucleotidyl transferase dUTP nick-end labeling
TLR4: Toll-like receptor 4
TEM: Transmission electron microscopy
TNF-α: Tumor necrosis factor-α
