# Supplementary material for: Pinocembrin ameliorates intermittent hypoxia-induced neuroinflammation through BNIP3-dependent mitophagy in a murine model of sleep apnea
Source: J Neuroinflammation. 2020 Nov 11;17:337. doi: 10.1186/s12974-020-02014-w (PMC7656728; doi:10.1186/s12974-020-02014-w)
Supplement: Supplementary file 1 — Additional file 1: Table S1. qRT-PCR primer sequences used in this study. [file 12974_2020_2014_MOESM1_ESM.docx]

**Table S1**

**qRT-PCR primer sequences used in this study**

| **Primer** |  | **Nucleotide sequence** |
| --- | --- | --- |
| GAPDH | Forward | 5'-ACCAGGTGGTCTCCTCTGAC-3' |
|  | Reverse | 5'-TGCTGTAGCCAAATTCGTTG-3' |
| TNF-α | Forward | 5'- CTCCTGGTATGAGATAGC-3' |
|  | Reverse | 5'- GTTGTACCTTGTCTACTCCC-3' |
| iNOS | Forward | 5'- AACATCAGGTCGGCCATCAC-3' |
|  | Reverse | 5'- AGCCTAGGTCGATGCACAAC-3' |
| COX-2 | Forward | 5'- AACCGAGTCGTTCTGCCAAT-3' |
|  | Reverse | 5'- CTAGGGAGGGGACTGCTCAT-3' |
| IL-6 | Forward | 5'- GGGACTGATGCTGGTGACAA-3' |
|  | Reverse | 5'- TCCACGATTTCCCAGAGAACA-3' |
| IL-1β | Forward | 5'- GGGCCTCAAAGGAAAGAATC-3' |
|  | Reverse | 5'- TACCAGTTGGGGAACTCTGC-3' |
