# Supplementary figures and images for: Pinocembrin ameliorates intermittent hypoxia-induced neuroinflammation through BNIP3-dependent mitophagy in a murine model of sleep apnea
Source: J Neuroinflammation. 2020 Nov 11;17:337. doi: 10.1186/s12974-020-02014-w (PMC7656728; doi:10.1186/s12974-020-02014-w)

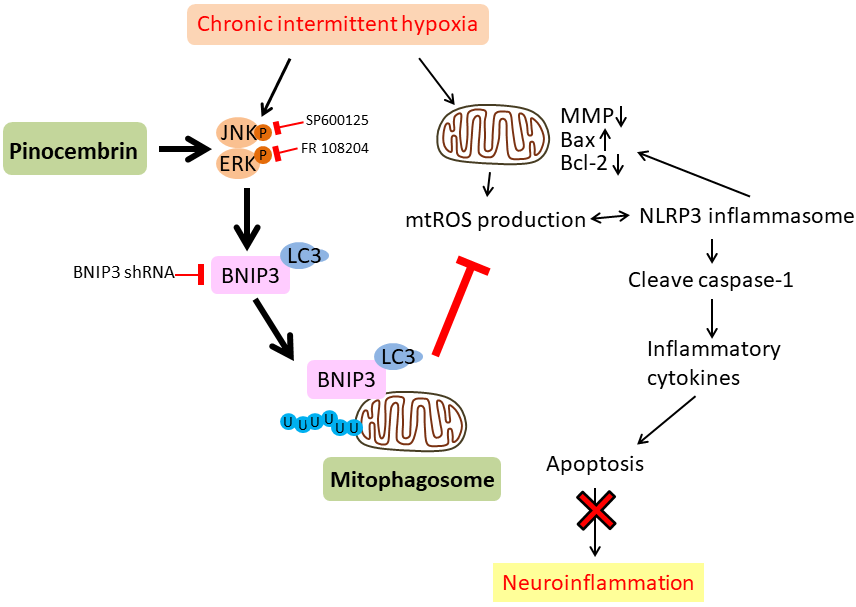

Supplement: Supplementary file 2 — Additional file 2: Figure S1. Pinocembrin protects against IH-induced neuroinflammation by upregulation of BNIP3-dependent mitophagy through JNK and ERK MAPK signalling pathways. [file 12974_2020_2014_MOESM2_ESM.tif]
